# Rapid detection of expanded short tandem repeats in personal genomics using hybrid sequencing

**DOI:** 10.1093/bioinformatics/btt647

**Published:** 2013-11-08

**Authors:** Koichiro Doi, Taku Monjo, Pham H. Hoang, Jun Yoshimura, Hideaki Yurino, Jun Mitsui, Hiroyuki Ishiura, Yuji Takahashi, Yaeko Ichikawa, Jun Goto, Shoji Tsuji, Shinichi Morishita

**Affiliations:** ^1^Department of Computational Biology, Graduate School of Frontier Sciences, The University of Tokyo, Chiba 277-8562, ^2^Department of Information and Communication Engineering, Faculty of Engineering and ^3^Department of Neurology, Graduate School of Medicine, The University of Tokyo, Tokyo 113-8655, Japan

## Abstract

**Motivation:** Long expansions of short tandem repeats (STRs), i.e. DNA repeats of 2–6 nt, are associated with some genetic diseases. Cost-efficient high-throughput sequencing can quickly produce billions of short reads that would be useful for uncovering disease-associated STRs. However, enumerating STRs in short reads remains largely unexplored because of the difficulty in elucidating STRs much longer than 100 bp, the typical length of short reads.

**Results:** We propose *ab initio* procedures for sensing and locating long STRs promptly by using the frequency distribution of all STRs and paired-end read information. We validated the reproducibility of this method using biological replicates and used it to locate an STR associated with a brain disease (SCA31). Subsequently, we sequenced this STR site in 11 SCA31 samples using SMRT^TM^ sequencing (Pacific Biosciences), determined 2.3–3.1 kb sequences at nucleotide resolution and revealed that (TGGAA)- and (TAAAATAGAA)-repeat expansions determined the instability of the repeat expansions associated with SCA31. Our method could also identify common STRs, (AAAG)- and (AAAAG)-repeat expansions, which are remarkably expanded at four positions in an SCA31 sample. This is the first proposed method for rapidly finding disease-associated long STRs in personal genomes using hybrid sequencing of short and long reads.

**Availability and implementation:** Our TRhist software is available at http://trhist.gi.k.u-tokyo.ac.jp/.

**Contact:** moris@cb.k.u-tokyo.ac.jp

**Supplementary information**: Supplementary data are available at *Bioinformatics* online.

## 1 INTRODUCTION

Many genetic disorders are caused by or associated with short tandem repeats (STRs), repetitive elements of 2–6 nt. Regarding the mechanism underlying the phenomenon of repeat expansion, unusual structural features of repeat-containing regions that affect cellular replication, repair and recombination are thought to induce frequent replication slippage, thereby expanding repeats (Mirkin, 2007). STRs have been found in a variety of genomic regions. Huntington’s disease is associated with expansion of the triplet repeat (CAG)*_n_* (polyglutamine runs in proteins) in the coding region of huntingtin (The Huntington's Disease Collaborative Research Group, 1993), where *n* < 28 in normal samples, *n* = 28–35 in intermediate cases, *n* = 36–40 in reduced penetrance and *n* > 40 in full penetrance (Walker, 2007). Spinal and bulbar muscular atrophy is also associated with (CAG) repeats in one exon (La Spada *et al.*, 1991).

In addition to exons, STRs have been observed in a variety of genomic regions such as untranslated regions (UTRs), introns and promoters. Fragile-X syndrome is associated with (CGG) repeat in the 5′-UTR (Kremer *et al.*, 1991; Sherman *et al.*, 1985; Verkerk *et al.*, 1991) and myotonic dystrophy type 1 (DM1) with (CTG) repeat in the 3′-UTR (Brook *et al.*, 1992; Mahadevan *et al.*, 1992). In introns, spinocerebellar ataxia type 10 (SCA10) is associated with (ATTCT) repeat (Matsuura *et al.*, 2000), myotonic dystrophy type 2 (DM2) with (CCTG) repeat (Liquori *et al.*, 2001), amyotrophic lateral sclerosis/frontotemporal dementia (ALS/FTD) with (GGGGCC) repeat (DeJesus-Hernandez *et al.*, 2011; Orr, 2011; Renton *et al.*, 2011) and SCA36 with (GGCCTG) repeat (Kobayashi *et al.*, 2011). Consequently, whole-genome sequencing capable of observing non-exonic regions is required to characterize STRs peculiar to a personal genome.

Several expanded repeats in RNA, such as CUG, CCUG, CAG, CGG, AUUCU and UGGAA, are associated with hereditary diseases and are known to accumulate in nuclear RNA foci in which several proteins are sequestrated in the process of foci formation (for a review see Wojciechowska and Krzyzosiak, 2011). These RNA foci are thought to have a negative effect on host cells, leading to disorders in cellular pathways (Wojciechowska and Krzyzosiak, 2011).

To search personal genomes for STRs, the most cost-efficient way would be to resequence an entire personal genome and to collect billions of short reads of ∼100 bp in length using available high-throughput sequencers. However, the infeasibility of obtaining longer reads at reasonable cost might lead to the failure to detect important STRs because expandable repeats associated with diseases can sometimes be quite long [e.g. (ATTCT)*_n_*, *n* = 800–4500 in SCA10 and (CCTG)*_n_*, *n* = ∼5000 in DM2] and are much longer than 100 bp, the typical length of short reads, making the identification and location of long STRs in a personal genome non-trivial.

Another serious problem is that STRs have several variants with many mutations. The spontaneous mutation rate of STRs, 3.78 × 10^−^^4^ to 7.44 × 10^−^^2^ in the human Y-chromosome (Ballantyne *et al.*, 2010), is far higher than the rate of copy number variation, 1.7 × 10^−^^6^ to 1.2 × 10^−^^4^ (Lupski, 2007), and the reported average rate of *de novo* single-nucleotide variation, 1.18 × 10^−^^8^ (SD = 0.15 × 10^−^^8^) (Conrad *et al.*, 2011) and 1.20 × 10^−^^8^ (Kong *et al.*, 2012). The ultrahigh mutation rate of STRs is thought to be a major force driving genetic variation producing a variety of STRs with differences often specific to personal genomes. Therefore, detecting various STRs by processing billions of short raw reads is fundamental to the analysis of personal genomes.

**Fig. 1.**
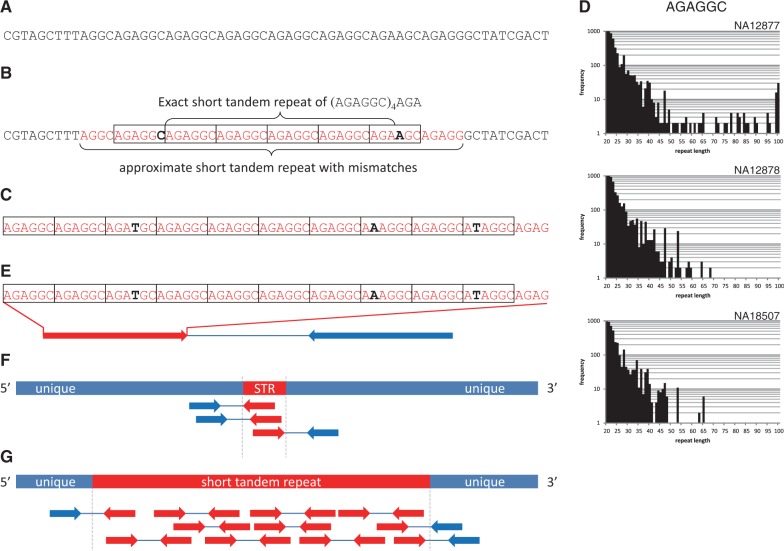
Sensing and locating STRs in short reads. (**A**) An original short read. (**B**) An approximate STR (AGAGGC)*n* (*n* = 6) in the short read. The central four copies of AGAGGC are an exact STR with no mutations, whereas the flanking copies contain the mutations shown in bold letters. If one of the regions (black) surrounding the STR aligns in a unique position, the STR can be located in the genome. (**C**) A read occupied by an approximate STR. (**D**) Sensing STRs from frequency distributions of (AGAGCC)*n* in NA12877 (father of the HapMap CEU trio), NA12878 (mother) and NA18507 (an African male). The *x*-axis is the lengths of STR occurrences detected in a read, and the *y*-axis is the frequency of reads containing STR occurrences of the length indicated on the *x*-axis. Note that 100-bp long STR occurrences are frequent in NA12877, whereas no STR occurrences of length >70 bp are observed in samples NA12878 and NA18507. (**E**) When a read is filled with an STR (red), we attempt to anchor the other end read (blue) to a unique position unambiguously. (**F** and **G**) An STR is located easily if its location can be sandwiched using information on paired-end reads. The length of an STR of length <100 bp is easily estimated (F), whereas determining the length of a much longer STR is non-trivial (G). We need to use third-generation sequencers, such as PacBio RS, with the capability of reading DNA fragments having a length of thousands of bases

Several software programs list STRs, such as Tandem Repeat Finder (Benson, 1999), Mreps (Kolpakov *et al.*, 2003), ATRHunter (Wexler *et al.*, 2005), IMEx (Mudunuri and Nagarajaram, 2007) and T-reks (Jorda and Kajava, 2009) (for a recent review that compares these programs, see Lim *et al.*, 2013); however, these conventional programs are designed to retrieve STRs from nearly complete or draft long genomes and are not intended for processing billions of short reads in a reasonable amount of time. Another problem involved in handling short reads is the difficulty of determining the accurate positions of STRs in the genome because reads filled with STRs are not included in the genome or often map to multiple locations. The problem is solvable in some cases when a flanking region around an STR in a read is long enough to map to a unique position (Fig. 1B). To resolve these special cases, Gymrek *et al.* developed the program lobSTR (Gymrek *et al.*, 2012), which improves the efficiency of this process by selecting ∼240 000 candidate regions harboring STRs in the human genome. Owing to severe restrictions in potential STR regions, however, we might overlook novel STRs hidden in numerous short reads because known STRs associated with diseases are frequently much longer than 100 bp, the typical length of short reads produced by high-throughput sequencers (Fig. 1C).

Here, we propose a new cost-efficient method for calculating a comprehensive collection of STRs that are longer than short reads by inspecting the frequency distribution of STRs in short reads. To approximate the locations of such STRs, we use paired-end sequencing to facilitate locating the opposite end of the read with the focal STR in a pair, thereby narrowing down the location of the focal STR. Finally, we present a statistical procedure for selecting STRs that are significantly expanded in the case sample.

## 2 METHODS

### 2.1 Non-redundant representation of STRs

Our goal was to enumerate all possible instances of STRs with 2–6-base-long repeat units efficiently. In general, our algorithm can detect repeat units of an arbitrary length without sacrificing computational time. We also present an example of disease-associated STRs with a 10-base repeat unit in SCA31 (Sato *et al.*, 2009). Care is required to avoid double counting identical STR occurrences characterized by more than one STR pattern. To remove redundancy, the basic unit of an STR should be minimized; e.g. the repeat unit of ACACACAC is AC rather than ACAC. Another reduction method is to merge occurrences of the reverse complement of an STR into the set of the focal STR. Therefore, we call the repeat unit representative if it is not a repeat of a shorter unit and is the first lexicographical motif when all possible shifts of the motif and its reverse complement are considered. Supplementary Table S1 presents the numbers of representative repeat units with typical examples.

### 2.2 Efficient algorithm for listing approximate STRs in billions of short reads

STRs are inherently ‘approximate’ in the sense that some unit occurrences are allowed to contain a small number of mutations (Ballantyne *et al.*, 2010). Listing approximate STRs, however, becomes computationally intractable because its time complexity grows exponentially in the maximum number of allowed mutations (Domanic and Preparata, 2007; Pellegrini *et al.*, 2010). Therefore, we use a heuristic approach to this problem. We first identify ‘exact’ STRs with no mutations in each short read using an efficient O(*n* log *n*)-time algorithm (Main, 1989), where *n* is the length of the read. A *repetition* is any non-empty string of the form 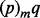
*,* where 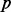, a non-empty string, is called the unit of the repetition, 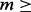 2, and 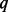 is a prefix of 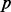. For example,

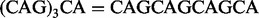

is a repetition of the form 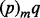
*,* where 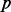 = CAG, 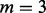 and 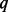 = CA, a prefix of 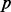. A repetition is *maximal* if it is not a proper substring of a repetition that has the same unit. For example, consider the following:

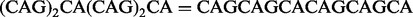



(CAG)_2_CA, a repetition with unit CAG, is maximal. In addition, the entire string is also a maximal repetition with unit (CAG)_2_CA. Listing all maximal repetitions is sufficient to identify all occurrences of STRs. We performed the following steps to retrieve STRs from each read.

1. Enumerate all maximal repetitions in a read using Main’s O(*n* log *n*)-time algorithm, where *n* is the length of the read (Main, 1989). More precisely, in 1984, Main and Lorentz designed an algorithm for enumerating all repetitions of the form *xx* (Main and Lorentz, 1984). In 1989, Main modified the algorithm to calculate maximal repetitions accurately (Main, 1989), and this is the version that we used to implement our system.
2. For each maximal repetition *Y*, identify the minimum unit *U* such that *U* is not a repetition and *Y* is a concatenation of multiple occurrences of *U* and a prefix of *U*. For example, when *Y* = (CAG) _6_CA, *U* = CAG.
3. An approximate repetition is a substring such that its alignment with repetition (*U*)_*m*_ is decomposed into series of exact matches of length |*U*| or more, and neighboring series must have only one mismatch, one insertion or one deletion between them in the alignment, where |*U*| indicates the length of *U*. We calculate an approximate repetition by extending a maximal (exact) repetition in both directions in a greedy manner. For example, given
  CGCCCGCAGCGCAT(CAG)_6_CATCAGGGA,
  we can extend repetition (CAG)_6_CA to the underlined substring,
  CGCCCGCAGC-GCA**T**(CAG)_6_CA**T**CAGGGA,
  where bold letters represent mismatches and ‘-’ indicates a deletion. In this way, we retrieve an approximate STR that is not necessarily an exact repeat of the minimum unit *U* but may contain mismatches and indels.
4. A read may contain multiple overlapping STRs with the same unit. If two overlap, eliminate the shorter one. If both are of the same length, select one arbitrarily.

The algorithm is able to process 10 million reads of length 100 bases in ∼1700 s on a Xeon X5690 with a clock rate of 3.47 GHz (Supplementary Fig. S1). As the computational time is proportional to the number of reads, ∼47 h is required to process 1 billion 100-bp reads, confirming the practicality of the method for processing real human resequencing data.

### 2.3 Sensing expanded STRs by analyzing the frequency distributions of STRs

The computational efficiency of our program facilitates the generation of frequency distributions of all approximate STRs in reads according to their lengths, as illustrated in Figure 1D. We used three samples of the whole-genome resequencing data downloaded from http://www.illumina.com/platinumgenomes/with accession numbers NA12877 (father of the HapMap CEU trio), NA12878 (mother) and NA18507 (an African male). We assumed that short reads were of length 100 bp, which is the typical length of reads output by cost-efficient high-throughput sequencers as of 2013. Although the length will likely increase in the near future, extending our procedure to process longer reads is straightforward because our algorithm runs in O(*n* log *n*)-time for processing reads of any length *n* as stated in the previous subsection. Comparing the distributions of more than one sample sometimes uncovers such a remarkable STR for which occurrences of length 100 bp are frequent in one sample (e.g. NA12877), but are absent in the other two samples, NA12878 and NA18507 (Fig. 1D), suggesting the presence of a long AGAGGC repeat in the former sample (Fig. 1D).

### 2.4 Reproducibility of detecting STR expansions for independent biological replicates

One might be concerned that despite the presence of a 100-bp long STR in a sample, our method might fail to report this with some probability. We examined this concern using two biological replicates collected independently from an identical DNA sample. The two replicates were independent datasets of 100-bp reads sequenced from the same DNA sample, NA12878, using an Illumina HiSeq2000 (Supplementary Table S2). One dataset was collected by DePristo *et al.* (2011) and the other dataset was downloaded from Illumina’s platinum genome Web site (http://www.illumina.com/platinumgenomes/). We applied our method to both biological replicates (Supplementary Table S2) and examined whether 100-bp occurrences of individual STRs were present simultaneously in both. We identified 60 STRs with 100 bp occurrences in one (*n* = 13, 21.7%) or both (*n* = 47, 78.3%) replicates of NA12878 (Supplementary Table S3). Of the 13 STRs with no counts in one replicate, 12 had one or two occurrences in the other replicate and the remaining one had four in the other. If an STR occurrence in the genome is short (e.g. 100 bp in length), failure to observe the STR has a high probability (e.g. 50% for 50-fold coverage of reads assuming the random collection of reads). Therefore, our method outputs essentially consistent results for the two biological replicates.

This analysis also indicated that the failure to detect 100-bp occurrences of an STR did not imply the absence of a 100-bp expansion of the STR in the focal personal genome. To be certain of its absence, we examined if the frequency distribution of lengths of STR occurrences was informative. Supplementary Figure S2 presents the frequency distributions of the 13 STRs in the two biological replicates. In most of the 13 STRs, when one biological replicate had 100-bp occurrences of an STR, the other replicate had occurrences of length >90 bp, although for two STRs, the longest occurrences were ∼60 bp, which might stem from factors such as amplification bias and variation in sequencing coverage. Therefore, the absence of >60-bp STR occurrences does not necessarily deny the existence of 100 bp expansions of the STR in the genome.

### 2.5 Locating long expansions of STRs in the human genome

The genomic positions of each uncovered STR in a read remain to be determined. The problem is solvable if one of the two regions flanking an STR maps to a unique position (Fig. 1B), the method used in lobSTR (Gymrek *et al.*, 2012). Otherwise, we attempt to use information on paired-end reads, the two ends of an identical DNA fragment such that their typical average length ranges from 300 to 350 bp with an average standard deviation of ∼10%. When one end-read is filled with an STR, we test whether the other end maps to a unique position in the genome using the Burrows–Wheeler Alignment Maximal Exact Matches algorithm (BWA-MEM), a tool for aligning reads with the genome (Li, 2013). If the test is successful, we can approximate the position of the STR from the location of the other end (Fig. 1E). An STR can be located if its location can be sandwiched using information on paired-end reads (Fig. 1F and G). An STR shorter than 100 bp is easier to determine (Fig. 1F), whereas estimating the lengths of longer STRs becomes more difficult (Fig. 1G). We will discuss this issue later in the text.

### 2.6 TRhist: a tool for sensing and locating STRs from billions of short reads

To assist in the correct positioning of STRs, for a read with an STR instance, our program outputs the repeat unit, length of the STR, number of mutations in the STR, flanking regions surrounding the STR and other paired-end read. With this information, the user can align the flanking regions and other end read to the reference to locate the STR in the genome. Our TRhist program is available at http://trhist.gi.k.u-tokyo.ac.jp/.

### 2.7 SMRT^TM^ sequencing of expanded STRs

Successful identification of an accurate position for one end provides useful input for other analytical methods, such as repeat-primed polymerase chain reaction (PCR) (Warner *et al.*, 1996) and SMRT^TM^ sequencing (Eid *et al.*, 2009; Loomis *et al.*, 2013), to estimate or determine long expansions of STRs. In particular, SMRT^TM^ sequencing is capable of reading DNA fragments of average length ∼5 kb as of 2013 (Fig. 1G). Using this emerging technology, Loomis *et al.* reported the first sequence, 750 CGG repeats, for fragile X syndrome (Loomis *et al.*, 2013). Using SMRT^TM^ sequencing, we amplified the repeat region associated with SCA31 using PCR primers 1.5k-ins-F (5′- ACTCCAACTGGGATGCAGTTTCTCAAT-3′) and 1.5k-ins-R (5′- TGGAGGAAGGAAATCAGGTCCCTAAAG-3′).

We will describe the analysis in the Section 3. PCR was performed in a final volume of 50 μl containing 0.2 μM of each primer, 200 μM of each dNTP, 1 mM MgCl2, 1.25 U of PrimeSTAR HS DNA polymerase (Takara Bio, Otsu, Japan) and 100 ng of genomic DNA. The PCR profile comprised an initial denaturing at 95°C for 5 min followed by 30 cycles at 95°C for 20 s and 68°C for 8 min. The PCR product was purified on 0.8% agarose gels and converted to the proprietary SMRTbell™ library format using an RS DNA Template Preparation Kit 2.0 (Pacific Biosciences, Menlo Park, CA). Briefly, the PCR product was end-repaired, and hairpin adapters were ligated using T4 DNA ligase. Incompletely formed SMRTbell^TM^ templates were degraded with a combination of exonuclease III and VII. The resulting DNA templates were purified using SPRI magnetic beads (AMPure; Agencourt Bioscience, Beverly, MA). Annealing was performed at a final template concentration of 5 nM, with a 20-fold molar excess of sequencing primer. The annealing reaction was carried out for 2 min at 80°C with slow cooling to 25°C. Annealed templates were stored at −20°C until polymerase binding. The DNA polymerase enzymes stably were bound to the primed sites of the annealed SMRTbell^TM^ templates using the DNA Polymerase Binding Kit 2.0 (Pacific Biosciences). SMRTbell^TM^ template (3 nM) was incubated with polymerase in the presence of phospholinked (Pacific Biosciences) nucleotides for 4 h at 30°C. Following incubation, the samples were stored at 4°C. Sequencing was performed within 36 h of binding. Samples were sequenced using commercial sequencing chemistry. Sequencing data were collected on a PacBio RS (Pacific Biosciences) for 90 min. Given PacBio RS-filtered subreads, we used the SMRT Pipe, P_ErrorCorrection module to generate corrected reads. Subsequently, we assembled these corrected reads using RS_CeleraAssembler to obtain contigs.

## 3 RESULTS

Here, we demonstrate the utility of an *ab initio* procedure for sensing, locating and sequencing STRs that are significantly expanded in the case sample.

**Locating candidate STR positions**

**Fig. 2.**
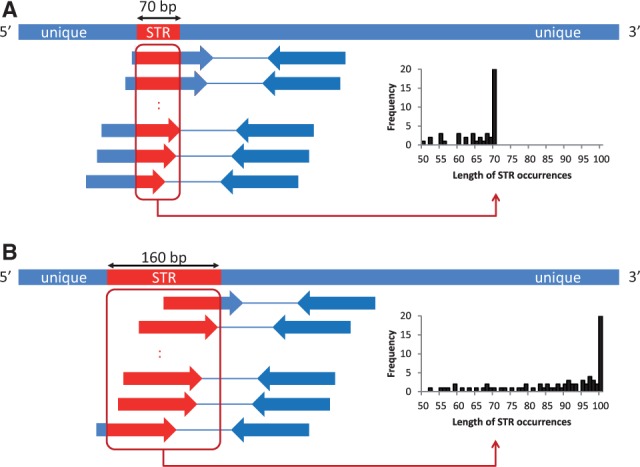
Select positions where STR occurrences are expanded significantly. (**A**) We generate the frequency distribution of lengths of STR occurrences in paired-end reads. This picture shows the case of a 70-bp long STR. The histogram of the frequency distribution peaks at 70 bp. (**B**) When the STR is 160-bp long, the distribution has a significant peak at 100 bp. We test if the peak is a significant outlier in the frequency distribution using the Smirnov–Grubbs’ test

Select positions where STR occurrences are expanded significantly in the case sample in the following manner:

1. Locate occurrences of each candidate STR in both the case and control samples by anchoring paired-end reads such that one end has a ≥50-bp occurrence of the STR and the other end maps to a unique position.
2. Group paired-end reads anchored in a neighborhood (within ∼300 bp, the average insert size of paired-end reads) into one cluster (Fig. 2).
3. In each cluster, generate the frequency distribution of STR occurrences according to their lengths ranging from 50 to 100 bp (Fig. 2). If an STR in the cluster is significantly longer than 100 bp, the frequency of 100-bp occurrences in reads, denoted by 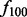, becomes significantly greater than the frequencies of those shorter than 100 bp (Fig. 2B). We test this hypothesis statistically by checking if 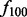 is an outlier in the frequency distribution with the Smirnov–Grubbs’ test. We calculate the *t*-score, 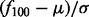, where 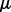 and 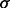 are the mean and standard deviation of the frequency distribution, respectively, and obtain the probability (*P*-value) that the *t*-score exceeds a threshold according to the Smirnov–Grubbs’ test. For example, the *P* < 5 × 10^−9^ when the *t*-score is >5.27.
4. We consider ∼10 million non-overlapping regions of length 300 bp (the average insert size of paired-end reads) in the human genome. We perform multiple hypothesis testing using the Bonferroni correction to test if each 300-bp region has a significant STR expansion in the case sample at a significance level of 5% divided by 10 million (i.e. 5 × 10^−9^). We select positions such that *P* < 5 × 10^−9^ in the case sample but no 100-bp STR occurrences are present in any of the control samples. We can relax the condition to consider more candidates with less evidence.

**Sequencing candidate STR positions**

SMRT^TM^ sequencing of expanded STRs is performed using information on the boundaries of individual STR positions.

### 3.1 A rare STR significantly expanded in the case sample

To demonstrate the effectiveness of this approach, we first examined a well-characterized case sample, SCA31 (Sato *et al.*, 2009), which contains long expansions of two STRs, (AAAATAGAAT) repeat and (AATGG) repeat, in the introns of genes BEAN1 and TK2 (Chr.16 66,524,303 in hg19), where the reference genome has an (AAAAT) repeat.

**Fig. 3.**
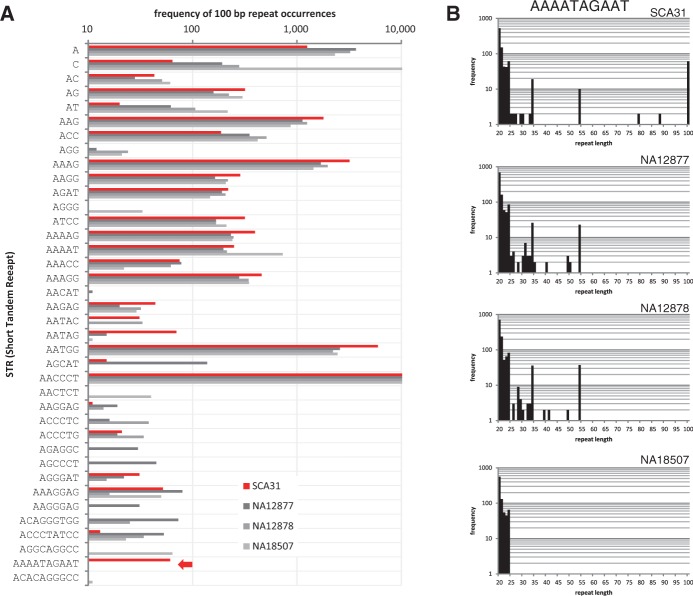
Sensing expanded STRs associated with SCA31. (**A**) Frequencies of 100-bp STRs that have >10 occurrences in one of SCA31, NA12877, NA12878 or NA18507. For example, the arrow in the second lowest row shows that the (AAAATAGAAT) repeat is expanded only in SCA31. Our *ab initio* procedure analyzes this bar chart and selects STRs that are significantly abundant in the case sample (e.g., SCA31) but absent in all of the control samples. The bar chart is also useful for confirming the abundance of (AATGG) and (AACCCT) repeats, equivalent to the (GGGTTA) repeat, where the former and latter motifs are known to be enriched in centromeres and telomeres, respectively. (**B**) Frequency distributions of the (AAAATAGAAT) repeat. SCA31 has many 100-bp occurrences, whereas no occurrences of length >55 bp were observed in NA12877, NA12878 and NA18507

We resequenced the genome of a sample from an individual whose parent is a case of SCA31 using an Illumina HiSeq2000 (Supplementary Table S4). All primary sequencing data of the SCA31 sample will be made available under controlled access through the DNA Databank of Japan (DDBJ; accession number JGAS00000000002). We examined whether we could find these STRs with no prior information. We applied the *ab initio* procedure to SCA31 as the case sample, and NA12877, NA12878 and NA18507 as control samples (Fig. 3A). Our procedure detected only one STR; AAAATAGAAT (*P* = 1.07 × 10^−19^).

Figure 3B shows the frequency distributions of the (AAAATAGAAT) repeat, supporting the presence of long occurrences of the STR in SCA31 and the absence of long occurrences of length >60 bp in the other control samples. Supplementary Figure S3A shows the distributions of the (AATGG) repeat, but the difference between SCA31 and the other samples was unclear because the (AATGG) repeat is enriched in human centromeres (Grady *et al.*, 1992). Therefore, our *ab initio* analysis suggests that long occurrences of the (AAAATAGAAT) repeat characterize SCA31, consistent with reported observations (Sato *et al.*, 2009). Arguably, we could detect the (AAAATAGAAT) repeat as an approximate (AAAAT) repeat because the last half, AGAAT, is identical to (AAAAT), except for the second base G; therefore, we analyzed the frequency distribution of the (AAAAT) repeat to determine the remarkable expansion of the (AAAAT) repeat in SCA31. This failed due to numerous long instances of the (AAAAT) repeat in all samples (Supplementary Fig. S3B). This example indicates the importance of looking at STRs of repeat units longer than 2–6-base units, to determine expansions of STRs associated with cases.

We also examined the frequency distributions of other well-characterized repeats, such as the (GGGTTA) repeat in telomeres (Supplementary Fig. S3C), (CAG) repeat encoding polyglutamine stretches in protein coding regions (La Spada *et al.*, 1991; The Huntington's Disease Collaborative Research Group, 1993; Walker, 2007 and Supplementary Fig. S4A), (CCTG) repeat associated with myotonic dystrophy type 2 (DM2; Liquori *et al.*, 2001 and Supplementary Fig. S4B) and (ATTCT) repeat associated with spinocerebellar ataxia type 10 (SCA10; Matsuura *et al.*, 2000 and Supplementary Fig. S4C). For the last three repeats, no significant differences were detected between SCA31 and the three control samples, suggesting that these three repeats are not associated with SCA31.

**Fig. 4.**
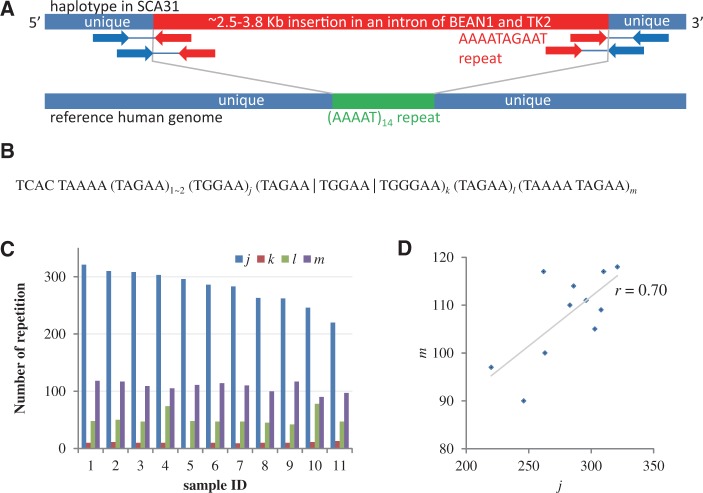
Locating and sequencing expanded STRs associated with SCA31. (**A**) A real example from SCA31. One haplotype contains a ∼2.5–3.8 kb insertion at Chr.16 66 524 303 in hg19 in an intron of BEAN1 and TK2. The right boundary of the insertion could be identified using paired-end reads with AAAATAGAAT repeats at their left ends and uniquely mapped reads at their right ends. The lower bar illustrates the reference genome (hg19) with an AAAAT repeat. (**B**) A form of expanded repeat associated with SCA31 samples. The values of *i*, *j*, *l* and *m* vary in the individual SCA31 samples. (**C**) We determined the values of *i*, *j*, *l* and *m* in 11 SCA31 samples using SMRT^TM^ sequencing. This shows that ∼90% of the repeat expansion are (TAGAA)*_j_* and (TAAAA TAGAA)*_m_*. (**D**) The values of ***j*** and ***m*** are positively correlated (*r* = 0.70). These two values are the determinants of the instability of the repeat expansions in SCA31

Using paired-end reads with AAAATAGAAT repeats at their 5′ ends and uniquely mapped reads at their 3′ ends, we could determine the 3′ end of the insertion. Figure 4A shows how we locate a ∼2.5–3.8 kb insertion of the repeat associated with the SCA31 sample (Sato *et al.*, 2009).

We sequenced the repeat region in 11 SCA31 samples using SMRT^TM^ sequencing. We designed a pair of PCR primers around the candidate repeat region in the SCA31 sample the right boundary of which could be determined. As illustrated in Figure 4A, we could identify the right boundary in the reference genome because the right ends of many paired-end reads mapped to the downstream region of the right boundary, whereas the left ends did not. We could sequence the candidate repeat region. Supplementary Table S6 presents the statistics of filtered subreads, corrected subreads and assembled contigs. Previously, Sato *et al.* estimated a 2.5–3.8 kb insertion of the following form for an SCA31 sample (Sato *et al.*, 2009):

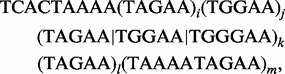

where (TAGAA | TGGAA | TGGGAA)*_k_* is a series of *k* occurrences of TAGAA, TGGAA and TGGGAA. In their sample, they determined that *i* = 2, *k* = 10 and *l* = 46, but left *j* and *m* undetermined because both appeared to be extremely long. In our 11 SCA31 samples, we could determine the values of *j* and *m*. We found that the numbers of individual repeats varied markedly (*i* = 1∼2, *j* = 220∼321, *k* = 9∼13, *l* = 42∼78 and *m* = 90∼118) and the insertion size ranged from 2350 to 3088 b (Fig. 4C and Supplementary Table S5), demonstrating the instability of the STR expansion in SCA31. In particular, two STRs, (TAGAA)*j* and (TAAAA TAGAA)*m*, form ∼90% of the entire repeat expansion, and the values of *j* and *m* are positively correlated (correlation coefficient *r* = 0.70), implying that these two values are the determinants of the instability of the repeat expansions in SCA31 (Fig. 4D). In all samples, the repeat expansion was present in one allele, but was absent in the other. Note that the numbers of STR units might not be exact because PCR for repeat regions can introduce more replication errors than those produced by bacterial DNA replication (Loomis *et al.*, 2013).

### 3.2 Common STRs significantly expanded in the case sample

**Fig. 5.**
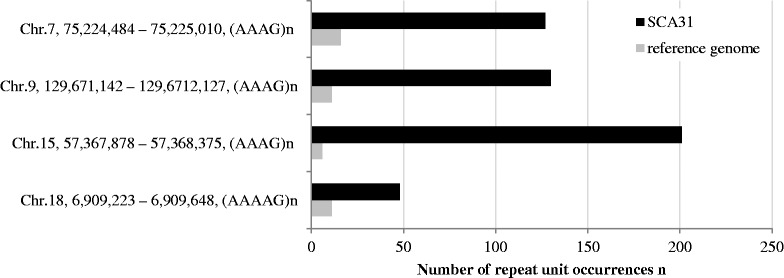
Sizes of the common STRs, (AAAG)*n* and (AAAAG)*n*, at four genomic positions in the SCA31 sample and reference genome. Note that individual STR occurrences are significantly expanded in the SCA31 sample. The PCR primers used for amplifying individual regions and the sequences of amplicons can be found in Supplementary Figure S6

We also applied our procedure to the SCA31 data, and examined common STRs, AAAG, ATCC, AAAAG, AATAG and AATGG, present in both the case and control samples but significantly expanded in the case sample. We identified STR expansions at 11 genomic locations that were significantly expanded in the case sample (*P* < 5 × 10^−19^ and Supplementary Fig. S5). We then used SMRT^TM^ sequencing to confirm the four expanded STRs in the case sample that were significantly longer than the corresponding STR occurrences in the reference genome (Fig. 5 and Supplementary Fig. S6). No false-positive expansions were found in this experiment, suggesting that the false-positive rate of the procedure is generally low.

## 4 DISCUSSION

STRs in personal genomes remain largely uncharacterized. We proposed a novel method for listing long approximate STRs with mutations in personal genomes using a massive number of short reads of length ∼100 bp. Here, we discuss some situations in which detecting a long expansion of STRs specific to disease samples is inherently problematic. As genomic regions of GC content >70% are difficult to cover with an ample number of Illumina reads, our method is unlikely to detect long expansions of STRs with high GC contents. STRs in reads originating in centromeres, telomeres or retrotransposons are too numerous to map to unique genomic positions. As illustrated in Supplementary Figure S3, massive numbers of long expansions of these STRs can be found in any sample.

We also presented an *ab initio* procedure for detecting significant expansions of STRs in case samples that are absent in control samples via comparisons between the frequency distributions of STRs in case and control samples. We demonstrated the potential applicability of this method using three publicly available control samples. To exploit this approach, however, constructing a large-scale database of the frequency distributions of STRs collected from a number of control samples is necessary.

The variety of expanded STRs of length >1 kb in disease remains unexplored. Also, examining whether expansions of STRs are more pronounced in germline and somatic cells would be intriguing. Thus, after locating STRs, sequencing expanded STRs is a promising direction of study. For this purpose, SMRT^TM^ sequencing enables the sequencing DNA fragments averaging ∼5 kb long as of 2013. Using SMRT^TM^ sequencing, we were able to determine a divergent set of 2.3–3.1 kb STR sequences in 11 SCA31 samples, showing the instability of STR expansions. Analysis of the stability of STR expansions in germline and somatic cells of a specific disease might eventually lead to the recognition of a functional role of STRs.

In the near future, the typical lengths of short reads in the majority of commercial sequencers should increase to 150–500 bases. Our method is ready to process longer reads in a straightforward manner. Furthermore, our method was designed so that it could output STRs of repeat units of any length, and we presented an illustrative case in which detecting STRs of a 10-base repeat unit from an SCA31 sample was essential. Our program will serve as a valuable tool for discovering unknown STRs in a variety of diseases, even with future advances in sequencing technology.

## Supplementary Material

Supplementary Data
