# Supplementary material for: Rapid detection of expanded short tandem repeats in personal genomics using hybrid sequencing
Source: Bioinformatics. 2013 Nov 8;30(6):815–22. doi: 10.1093/bioinformatics/btt647 (PMC3957077; doi:10.1093/bioinformatics/btt647)
Supplement: Supplementary Data [file supp_30_6_815__index.html]

Rapid detection of expanded short tandem repeats in personal genomics using hybrid sequencing — Rapid detection of expanded short tandem repeats in personal genomics using hybrid sequencing — Rapid detection of expanded short tandem repeats in personal genomics using hybrid sequencing — Supplementary Data 

# Rapid detection of expanded short tandem repeats in personal genomics using hybrid sequencing

## Supplementary Data

files

**Files in this Data Supplement:**

- Supplementary Data - pdf file
