# Supplementary material for: Rapid detection of expanded short tandem repeats in personal genomics using hybrid sequencing
Source: Bioinformatics. 2013 Nov 8;30(6):815–22. doi: 10.1093/bioinformatics/btt647 (PMC3957077; doi:10.1093/bioinformatics/btt647)
Supplement: Supplementary Data [file supp_btt647_supplementary_figures_revision_2.pdf]

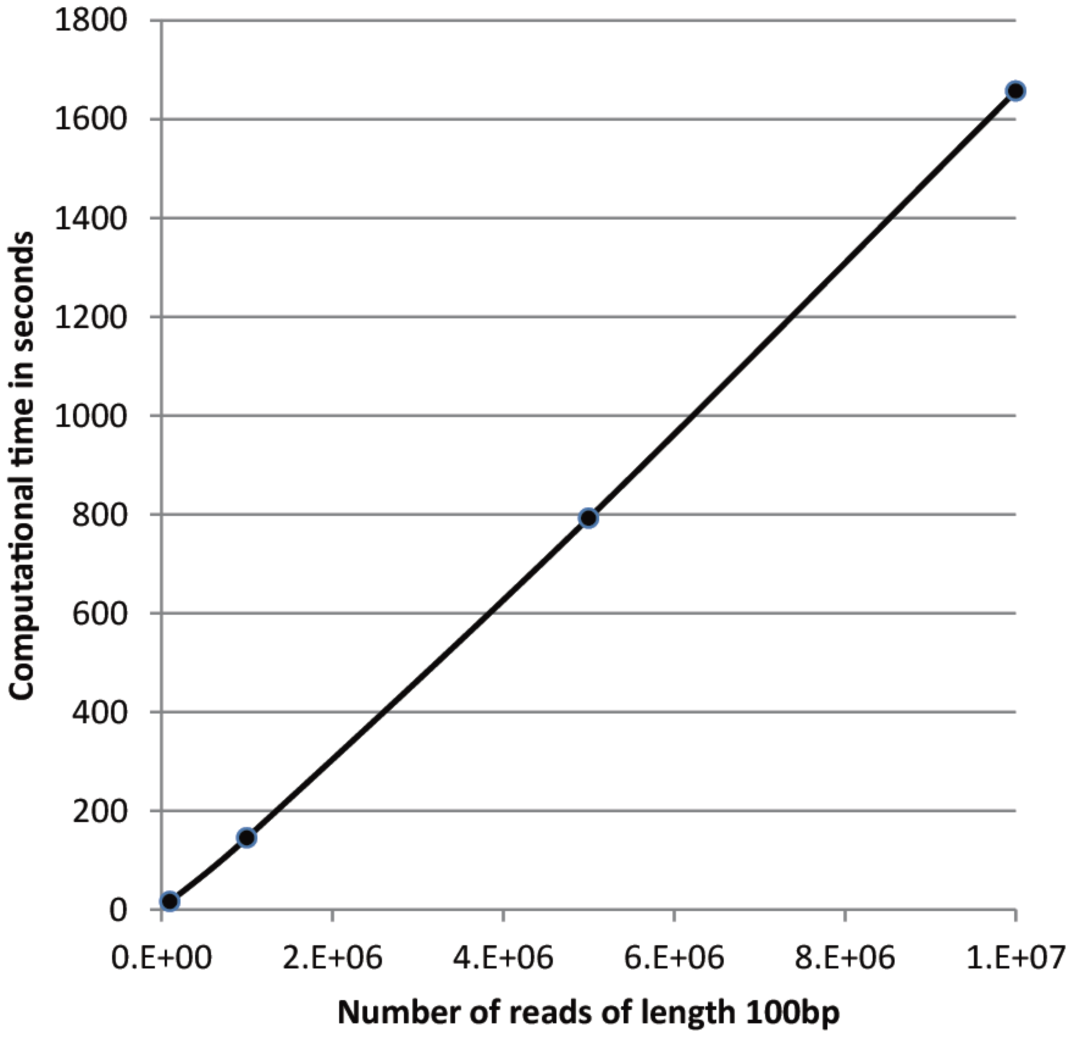

Supplementary Figure S1. Computational Performance of the algorithm

This graph shows computational time to retrieve short tandem repeats from 100bp reads using Xeon X5690 with a clock rate of 3.47GHz. Our algorithm implemented in Java needs 200-300MB of the main memory.

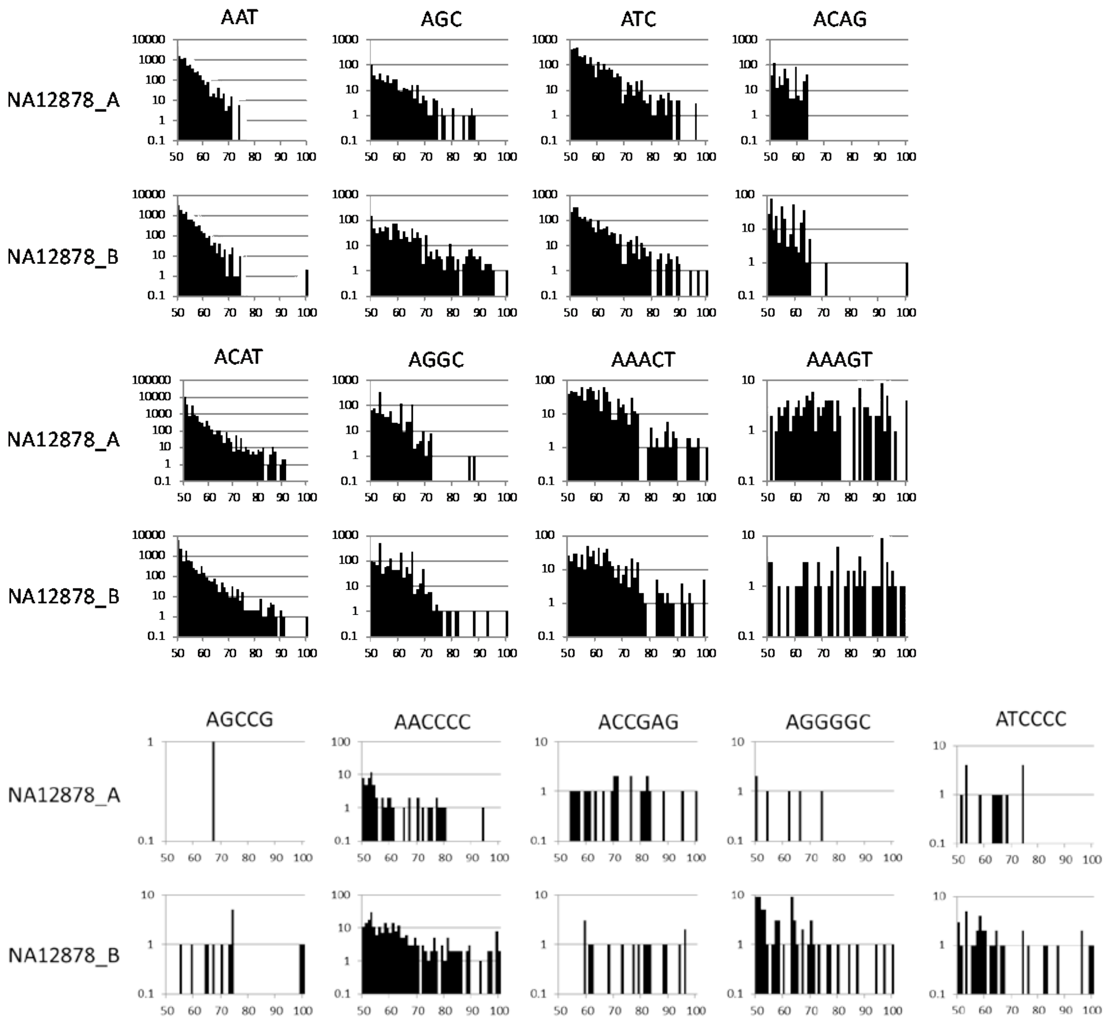

Supplementary Figure S2. Frequency distribution of occurrences of 13 STRs that had 100bp occurrences in one of the two biological replicates collected from NA12878, where one replicate was collected by DePristo *et al.* (DePristo, et al., 2011) and the other dataset was downloaded from http://www.illumina.com/platinumgenomes/. We call the former NA12878_A and the latter NA12878_B. Note that the presence of 60-70bp occurrences of STRs, such as AAT, ACAG, and AGCCG, in one replicate may imply the existence of 100bp occurrences of the STR in the other replicate.

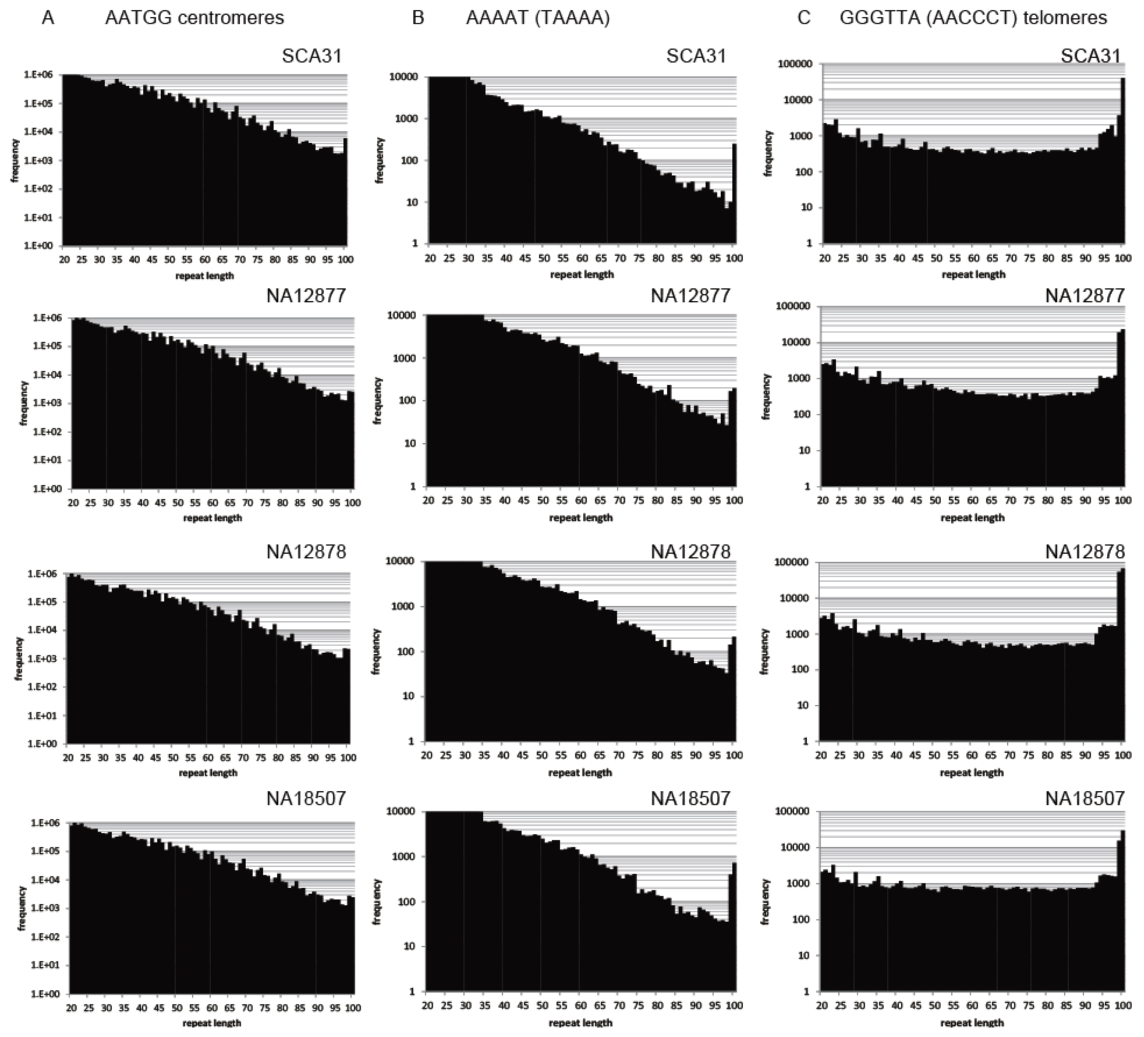

Supplementary Figure S3. Frequency distributions of STRs in SCA31, NA12877, NA12878, and NA18507.

A. Distributions of (AATGG) repeat that is enriched in human centromeres.

B. Distributions of (AAAAT) repeat are similar among the four samples. As discussed in the main text, one might argue that we could detect (AAAATAGAAT) repeat as an approximate (AAAAT) repeat because the last half, AGAAT, is identical to AAAAT except for the second base G, motivating us to analyze the frequency distribution of (AAAAT) repeat to uncover a remarkable expansion of (AAAAT) repeat in SCA31. This attempt failed because of numerous instances of (AAAAT) repeat in all the samples.

C. Distributions of (AACCCT) repeat that is enriched in human telomeres. Indeed, in all of the samples, we observed a massive number of 100bp reads filled with (AACCCT) repeat.

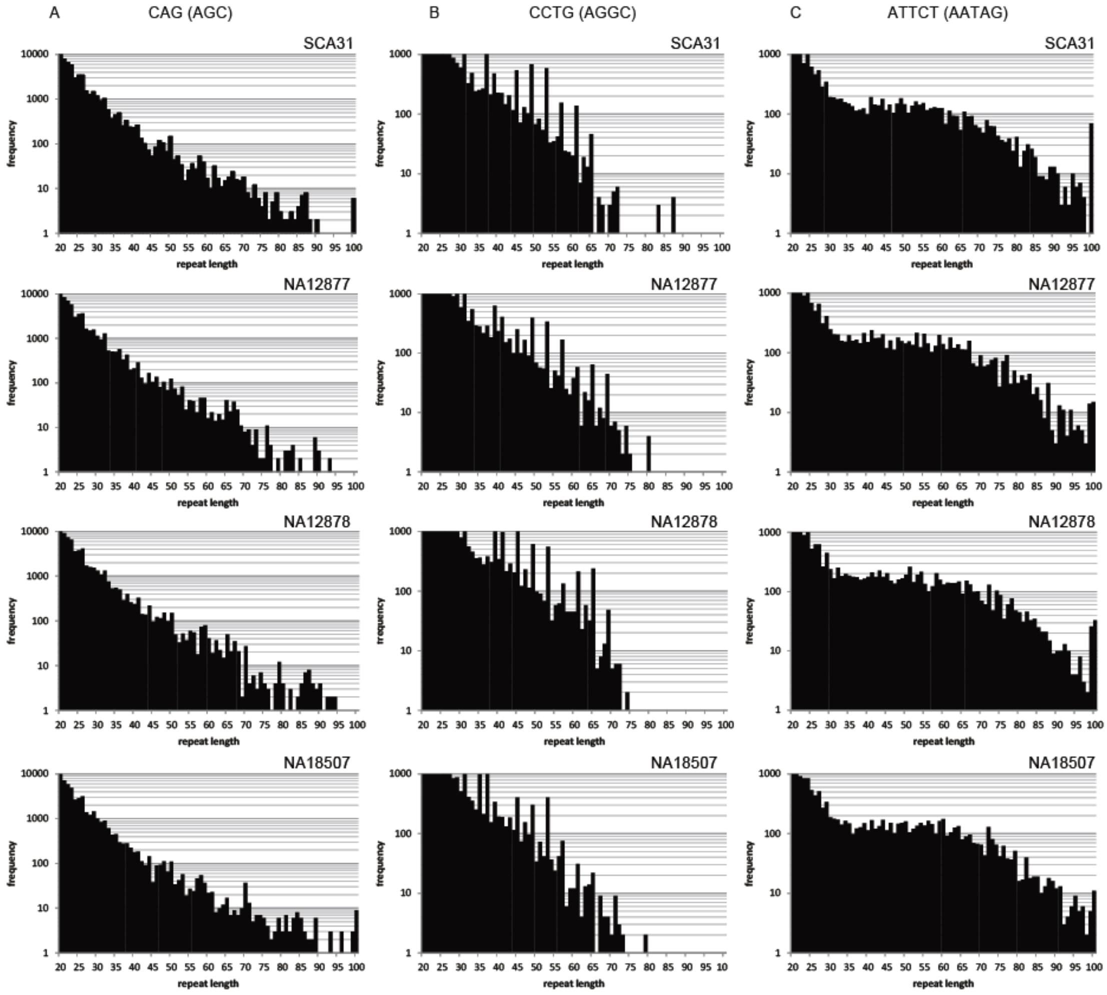

Supplementary Figure S4. Frequency distributions of STRs associated with disease in SCA31, NA12877, NA12878, and NA18507.

A. Distributions of (CAG) repeat encoding polyglutamine in protein coding regions.

B. Distributions of (CCTG) repeat associated with spinocerebellar ataxia type 10 (SCA10). Distributions of each STR are similar among the four samples, suggesting that the three repeats are not associated with SCA31.

A

STR unit	chr	position	Number of aligned reads at boundaries<br>left	right	t-score	p-value
AAAAG	chr5	78,475,851	3	8	5.86	4.82E-13
	chr18	6,908,890	29	14	6.91	2.29E-39
AAAG	chr1	18,829,023	1	11	5.88	3.45E-13
	chr2	10,946,441	7	4	6.61	8.12E-24
	chr4	128,469,155	3	6	6.17	4.14E-16
	chr6	24,056,283	11	8	6.34	1.85E-18
	chr7	75,224,173	32	5	6.80	4.76E-31
	chr9	129,671,056	27	16	6.75	2.70E-28
	chr15	57,367,589	22	9	6.22	8.91E-17
AATAG	chr21	36,720,550	6	9	5.70	8.81E-12
AATGG	chr16	66,523,972	10	0	6.59	3.22E-23

B

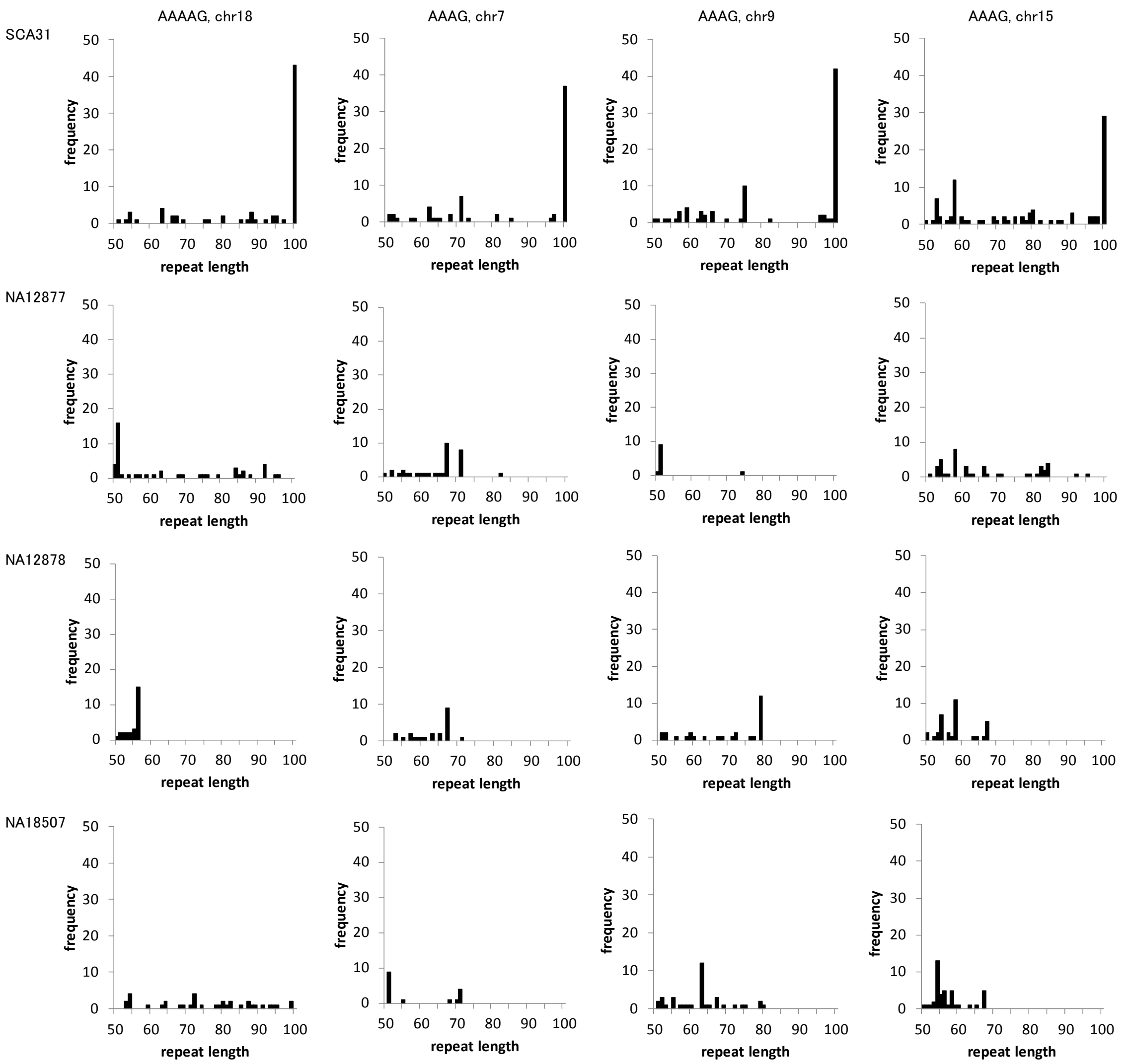

Supplementary Figure S5. Common STRs significantly expanded in the SCA31 sample.

A. We associate individual occurrences of STRs located in eleven genomic regions with their t-scores and p-values according to Smirnov-Grubbs' test. The table displays the number of aligned reads at the left and right boundaries. We selected four regions (colored red) such that t-score > 6 and the total of aligned reads > 30 for further PacBio sequencing.

B. In each of the four selected regions, we generate the frequency distribution of STR occurrences according to their lengths ranging from 50 b to 100 b for the case SCA31 sample and three control samples, NA12877, NA12878, and NA18507.

A

PCR amplified genomic region	Size (bp)	STR	Number of occurrences		PCR primers	
			reference genome	SCA31	forward	reverse
Chr.7, 75,224,484 – 75,225,010	990	(AAAG)n	16	127	5'-ATCAGCTTTTGGTGTAGGAG-3'	5'-CCAGAAAGGCATTATTTCTG-3'
Chr.9, 129,671,142 – 129,6712,127	850	(AAAG)n	11	130	5'-GAGCAGGAGCTACCTCTTAG-3'	5'-TGGGAAGGTTCAAGGAGTTG-3'
Chr.15, 57,367,878 – 57,368,375	1260	(AAAG)n	6	201	5'-ATTTAAGCCTGAGTTCTCTC-3'	5'-ACACATTAGAAATCTGATGC-3'
Chr.18, 6,909,223 – 6,909,648	622	(AAAAG)n	11	48	5'-AATGTTGATATGGAGTAGGC-3'	5'-TTGCAGAGTCTCCAGAGAG-3'

B

Chr.7, 75,224,484 – 75,225,010

```
ATCAGCTTTTGGTGTAGGAGTCCTTTCTTTCTTTCTTTCTTTCTTTCTTTCTTTCTTCTTTCTTCTTTTTTCTTTCTTTCTTTCTTTCTTTCTTTCTTTC
TTTCTTTCTTTCTTTCTTTCTTTCCTTTCTTTCTTTCTTTCTTTCTTTCTTTCTTTCTTTCTTTCTTTCTTTCTTCTTTCTTTCTTTCTTTCTTTCTTTC
TTTCTTTCTTTCTTTCTTTCTTTCTTTCTTTCTTTCTTTCTTCTTTCTTTCTTTCTTTCTTTCTTCTTTCTCTTTCTTTCTTTCTTTCTTTCTTTCTTTC
TTTCTTTCTTTCTTTCTTTCTTTCTTTCTTTCTTTCTTTCTTTCTTTCTTTCTTTCTTTCTTTCTTTCTTTCTTTCTTTCTTTCTTTCTTCTTTCT
TTCTTTCTTTCTTTCTTTCTTTCTTTCTTTCTTTCTTTCTTTCTTTCTTTCTTCTTTCTTCCTTTCTTTCTTTCTTTCTTTCTTTCTTTTCTTTCTTCTT
TCTTTCTTTCTTTCTTTCTTTTTTTTTTTTTTTTTTTCTTTTTTTTCTTTTTTTTCTTCTTTCCTTCCTTCCTTCCTTCTTTCTTTCTTTTTTTTCTTTCTCT
CTCTTCCTTCCTTCCTCCCCTCTCTCTCCTCGTTCGTTCTTTCTTTCTATTTTTTTTTTAGACAGGGTCTCGCTCTGTTGCCCAGGCTGGAGTGCAGTGG
CACAATCTTGGCTCACTGCAACCTCCACCTCCCAGGTTCAAGTGATTCTCGTGCCTCAGCCTCCCAAGTAGCTGGGATCACAGGCACCCGCCACCATGCC
CGGCTAATTTTTGTTTTTTTTAGTAGAGACGGGGTTTCACCATGATGCTCAGGCTGGTCTCGAACTCCTGGCCTCAAGAGATCCACCCACCTTGGCCTCC
CAAAGTTCTGGGATTACAGGTGTGAGCCACTGTGCCTGGCCTTGGTGTAGGAGCGATTTCTGAAATAATCCAGAAATAATGCCTTTCTGG
```

Chr.9, 129,671,142 – 129,6712,127

```
GAGCAGGAGCTACCTCTTAGGCAACAGAAAAGAGTGGGTTATGGCCTGTATTAGTTATCTTTTGCTGTGTAACAATTTTATCCCAAATGTGGCAGCTTAA
AATAACACACATTTATTATCTCAATGTTTCTATAGGTTAGGGACAGCTTTGCTAGGCCCTCTTCTTCAGAGTCTCACAGGCTATAATCAAGGTGTTGGCT
GTGGCTGCAGTCACATCTGAGGCTCAACAGTTGAAGGATCTGCTTCTGAGCTCATGTGATTATTGGCAGCATTCTGTTCCTTTCAGGCTGCTGGGCTGAG
GGCCCCAGATTCATTGTTTCTTTTCTCTTTCTTTCTTTCTTTCTTTCTTTCTTTCTTTTCTTTCTTTCTTTCTTTCTTTCTTTCTTTCTTTCTTTCTTTC
TTTCTTTCTTTCTTTCTTTCTTTCTTTCTTTCTTTCTTTCTTTCTTTCTTTCTTTCTTTCTTTCTTTCTTTCTTTCTTTCTTTCTTTCTTTCTTTCTTTC
TTTCTTTCTTTCTTTCTTTCTTTCTTTCTTTTCTTTCTTTCTTTCTTTCTTTCTTTCTTTCTTTCTTTCTTTCTTTCTTTCTTTCTTTCTTTCTTTCTTT
CTTTCTTTCTTTCTTTCTTTCTTTCTTTTCTTTCTTTCTTTCTTTCTTTCTTTCTTTCTTCTTCTTTTCTTTCTTTCTTTCTTTCTTTCTTTCTTT
CTTTCTTTCTTTCTTTCTTTCTTTCTTTCTTTCTTTCTTTCTTTCTTTCTTTCTTTCTTTCTTTCTTTTCCTTTCTTTCTGTTCTTTCTTTTCTTTCTTT
CTTTCTTTCTTTTCTTATCTTTCTTTCTTTCTTCTTTCTTTCCTGAGACG
```

Chr.15, 57,367,878 – 57,368,375

```
ATTTAAGCCTGAGTTCTCTCATTAGACAAATGAACTTGGTCAAATGGGTCATTTTTTCTGGACATGACATGAGGTTTCTTTTCTTTCTTTCTTTCTTTCT
TCTCTTTCTTTCTTTCTTTCTTTCTTTCTTTCTCTCTCTCTTCTCTCTCTCTTTCTCTCTTTCTCTTTCTTTCTTTCTTTCTTTCTTTCTTTCTTCTTTC
TTTCTTTCTTTCTTTCTTTCTTTCTTTTCTTTTCTTCTTCTTTCTTTCTTTCTTTCTTTCTTTCTTTCTTTCTTTCTTTCTTTCTTTCTTTCTTTCTTTC
TTTCTTTCTTTCTTTCTTTCTTCTTTCTTTCTTTTCTTTCTTTCTTTCTTTCTTTCTTTCTTTCTTTCTTTCTTTCTTTCTTTCTTTCTTTCTTTCTTTC
TTCTCTTCTTTCTTTCTTTCTTTCTTTCTTTCTTTCTCTTCTTTCTTTCTTTCTTTCTTTCTTTCTTTCTTTCTTTCTTTCTTTCTTTCTTTCTTTCTTT
TCTTTCTTTCTTTCTTCTTTCTTTCTTCTTTCTTTCTTTCTTCTTTCTTTCTTTCTTTCTTTCTTCTTTCTTTCTTTCTTTCTTTCTTTCTTTCTTTCTT
TCTTTCTTTCTTTCTTTCTTTTCTTTCTTTCTTTCTTTCTTTTCTTTCTTTCTTTCTTTCTTTCTTCCTTTCTTTCTTTCTTTCTTTCTTTCTTTCTTTC
TTTCTTTCTTTCTTTCTTTTCTTTCTTTCTTTCTTTCTTCTTCTTTCTTTCTTTCTTTCTTTCTTTCTTTTCTTTCTTCTTTCTTTCTTTCTTTCTTTCT
TTCTTCTTTCTTTCTTTCTTTCTTTCCTTTCTTTCTTTCTTTCTTTCTTTCTTTCTTTCTTCTTTCTTTCTTTCTTTCTTCTTCTTTTCTTTCTTTCTTT
CTTTCTTTCTTTCTTTCTTTCTTTCTTTCTTTCTTTCTTTCTATTCTTTTTTTTTATTTGAGACAGGGTCTTTCTCTGTTGCCCAGGCTAGAGTACAGTG
ACACGATCGTGGCCTATTGCAGCATTGAACCCCTGGGTTCAGGTGATCCTCTGCCCCTGTCTCCCAAGTAGCTGGGACTACAGGCCTGTGCTACCATATC
TGGCTAATTTTTTGTAGAGACAGGGTCTCACTTAGGTTGCCCAGGCTGTTACCAAATTCCTGAGCTCAAGGTGATCCTCCTGCCTGGCCTCCCAAAGTCC
TGAGATAATATAGGCATGAGCCACTGCGTTCGGTGAATGGGCATCAGATTTCTAATGTGT
```

Chr.18, 6,909,223 – 6,909,648

```
AATGTTAATGTTGATATGGAGTAGGCTTGGGTCTTTTCTTTTCTTTTCTTTTCTTTTCTTTTCTTTTCTTTTCTTTTCTTTTCTTTTCTTTTCTTTTCTT
TTCTTTTCTTTTCTTTTCTTTTCTTTTCTTTTCTTTTCTTTTCTTTTCTTTTCTTTTCTTTTCTTTTCTTTTCTTTTCTTTTCTTTTCTTTTCTTTTCTT
TTCTTTTCTTTTCTTTTCTTTTCTTTTCTTTTCTTTTCTTTTCTTTTCTTTTTCTTTTCTTTTCTTTTCTTTTTTTTTTTTTGAGACAGAATCTTTCTC
TGTTGCCCAGGTTGGAGTGCAGTGGAGTGATCTTGGCTCACTGCAACCTCCGCCTCCCGGGTTCAAGTGATTCTCCTGCCTCAGCCTCCCTAGTAGCTGG
GATTGCAGATGCCCGCCACCACGCCCAGACAATTTTTATATTTTTAGTAGAGACGGGGTTTCACCATGTTGGCCAGGCTGGTCTCGAACTCCTGACCTCA
AGTGATCCACCCGCCTCGGCCTCCCAAGGTGCTGGGATTACAGGTGTGAGCCACTGCGCCTGGCTGGCTTCGGTCTTTTCAGTGTGATTTGAAGTTGGAA
AGTCTCTCTGGAGACTCTGCAA
```

Supplementary Figure S6. Sequencing common STRs at four genomic positions.

A. The table shows four PCR amplified regions with their sizes (bp), STRs in individual positions, numbers of repeat unit occurrences (*n*) in the reference genome and the SCA31 sample, and forward and reverse PCR primers for amplifying each region. Note that STR occurrences in the SCA31 sample are remarkably expanded.

B. The sequences of the amplicons in the four regions that are determined using SMRT™ sequencing. Red colored substrings show STR occurrences. Each line contains 100bp. The reverse complement of STR (CTTT)*n* gives (AAAG)*n.*

Supplementary Table S1. Numbers of representative units. A unit is representative if it is not a repeat of a shorter unit and is the first lexicographical motif when all possible shifts of the motif and its reverse complement are considered. The table shows the numbers of representative units of length 1-10 nt and typical examples of representative units.

Unit length	Number of representative units	Examples of representative units
1	2	A, C
2	4	AC, AG, AT, CG
3	10	AAC, AAG, AAT, ACC, ACG, ACT, AGC, AGG, ATC, CCG
4	33	AAAC, AAAG, AAAT, AACC, AACG, AACT, AAGC, AAGG, AAGT, AATC, AATG, AATT, ACAG, ACAT, ACCC, ACCG, ACCT, ACGC, ACGG, ACGT, ACTC, ACTG, AGAT, AGCC, AGCG, AGCT, AGGC, AGGG, ATCC, ATCG, ATGC, CCCG, CCGG
5	102	AAAAC, AAAAG, AAAAT, AAACC, …
6	350	AAAAAC, AAAAAG, AAAAAT, AAAACC, …
7	1,170	AAAAAAC, AAAAAAG, AAAAAAT, …
8	4,173	AAAAAAAC, AAAAAAAG, AAAAAAAT, …
9	14,560	AAAAAAAAC, AAAAAAAAG, AAAAAAAAT, …
10	52,734	AAAAAAAAAC, AAAAAAAAAG, AAAAAAAAAT, …

Supplementary Table S2. Statistics for two biological replicates collected from NA12878. One replicate was collected by DePristo *et al.* (DePristo, et al., 2011) and the other dataset was downloaded from Illumina's platinum genome web site (http://www.illumina.com/platinumgenomes/). We call the former NA12878_A and the latter NA12878_B.

	NA12878_A	NA12878_B
Number of reads	2,499,770,346	1,708,169,546
coverage	83.3	56.9

Supplementary Table S3. Numbers of 100bp Illumina reads that are filled with occurrences of common STRs of unit length 2-6 bp in two biological replicates NA12878_A collected by DePristo *et al.* (DePristo, et al., 2011) and NA12878_B downloaded from Illumina's platinum genome web site. The two biological replicates are denoted by A and B respectively in the tables. STRs with no counts in both replicates are not included in the tables. The middle and rightmost tables show 47 STRs such that their 100bp occurrences are observed in both replicates, the leftmost table shows 13 STRs with no counts in one of the two replicates. Although the leftmost table presents a discrepancy between the two biological replicates, the difference is small because 12 of 13 STRs have only one or two occurrence in one replicate and none in another.

STR	A	B
AAT	**0**	2
AGC	**0**	1
ATC	**0**	1
ACAG	**0**	1
ACAT	**0**	1
AGGC	**0**	1
AAACT	1	**0**
AAAGT	4	**0**
AGCCG	**0**	1
AACCCC	**0**	2
ACCGAG	1	**0**
AGGGGC	**0**	1
ATCCCC	**0**	1

STR	A	B
AC	117	51
AG	656	223
AT	29	106
AAG	2612	1251
ACC	635	510
AGG	55	24
AAAG	4501	1969
AACC	20	1
AAGG	534	218
ACTC	3	2
AGAT	428	207
AGGG	4	2
ATCC	432	168
AAAAG	500	246
AAAAT	90	213
AAACC	152	62
AAAGG	919	344
AAATG	9	2
AACAT	17	5
AAGAG	58	32
AAGGG	3	1
AATAC	4	1
AATAG	49	33
AATAT	1	2

STR	A	B
AATGG	5294	2212
ACAGC	5	2
ACATC	16	8
ACCAT	3	6
AGAGG	2	1
AGCCC	6	7
AGGGC	5	3
ATCCC	6	2
AACCCG	4	10
AACCCT	135330	68247
AACTCT	4	2
AAGGAG	38	14
ACATAT	11	6
ACATCC	2	1
ACCATC	6	3
ACCCTC	56	38
ACCCTG	27	34
ACCTAT	3	2
AGAGAT	3	2
AGAGGC	2	1
AGAGGG	3	2
AGCCCT	4	2
AGGGAT	30	15

Supplementary Table S4. Statistics of 100bp paired-end reads collected from SCA31 using Illumina HiSeq2000.

# Read	#Mapped read	#Mapped read (unique)	Mapping rate, %	Mapping rate, % (unique)	Coverage	Coverage (unique)
1,627,990,590	1,557,034,174	1,444,151,887	95.6	88.7	50.3	46.65

Supplementary Table S5. Structure of STR in SCA31. The table presents the values (numbers of repetition) of *i, j, k, l, m* of five repeat units of the following STR form in eleven SCA31 samples:

TCAC TAAAA $(TAGAA)_i$ $(TGGAA)_j$ $(TAGAA \mid TGGAA \mid TGGGAA)_k$ $(TAGAA)_l$ $(TAAAA\ TAGAA)_m$

The values of *j* and *m* are correlated (correlation coefficient $r = 0.70$, $n = 11$).

sample ID		1	2	3	4	5	6	7	8	9	10	11
(TAGAA) *i*		1	1	2	1	1	1	2	1	1	2	1
(TGGAA) *j*		308	283	262	296	286	310	246	263	321	220	303
(TAGAA | TGGAA | TGGGAA) *k*	*k*	10	9	10	0	10	11	11	10	10	13	10
	occurrence	TGGGAA<br>TAGAA<br>TGGGAA<br>(TAGAA)2<br>(TGGAA)2<br>TAGAA<br>(TGGAA)2	TAGAA<br>TGGAA<br>(TAGAA)2<br>(TGGAA)2<br>TAGAA<br>(TGGAA)2	TGGGAA<br>TAGAA<br>TGGGAA<br>(TAGAA)2<br>(TGGAA)2<br>(TAGAA)2<br>TGGAA	none	TGGGAA<br>TAGAA<br>TGGAA<br>(TAGAA)2<br>(TGGAA)2<br>(TAGAA)2<br>TGGAA	TAGAA<br>(TGGAA)2<br>TAGAA<br>(TGGAA)2<br>TAGAA<br>(TGGAA)2<br>TAGAA<br>TGGAA	TAGAA<br>(TGGAA)2<br>TAGAA<br>(TGGAA)2<br>TAGAA<br>(TGGAA)2<br>TAGAA<br>TGGAA	TGGGAA<br>TGGAA<br>(TAGAA)2<br>TGGAA<br>TAGAA<br>(TGGAA)4	TAGAA<br>TGGAA<br>(TAGAA)2<br>(TGGAA)2<br>(TAGAA)2<br>(TGGAA)2	(TAGAA)3<br>TGGGAA<br>TAGAA<br>TGGAA<br>(TAGAA)2<br>(TGGAA)2<br>(TAGAA)2<br>TGGAA	TAGAA<br>(TGGAA)2<br>(TAGAA)2<br>(TGGAA)2<br>(TAGAA)2<br>TGGAA
(TAGAA) *l*		47	47	42	48	47	50	78	45	48	47	74
(TAAAATAGAA)*m*		109	110	117	111	114	117	90	100	118	97	105
total length (bp)		2930	2813	2770	2839	2870	3041	2596	2602	3088	2350	2999

Supplementary Table S6. Statistics (number, average length, and maximum length) of filtered raw subreads, corrected subreads, and assembled contigs of eleven samples.

sample ID	1	2	3	4	5	6	7	8	9	10	11
Raw subreads											
Number	42,085	122,522	51,508	88,163	8,941	41,714	29,890	27,144	45,724	31,916	29,049
Average length	1,370	807	1,731	1,036	1,021	2,044	2,264	2,019	1,940	2,078	1,934
Maximum length	12,916	15,540	15,791	15,777	10,367	19,273	16,508	17,787	16,964	16,004	14,891
Corrected subreads											
Number	2,885	7,250	3,098	7,867	1,070	1,919	1,313	1,354	2,344	1,543	1,816
Average length	864	1,250	1,483	1,258	1,282	1,540	1,583	1,443	1,501	1,509	1,384
Maximum length	4,577	3,264	4,233	3,927	3,327	4,450	4,730	3,346	4,572	3,456	4,497
Assembled reads											
Number	8	10	7	43	1	3	5	7	6	7	11
Average length	2,993	2,005	2,542	1,921	4,401	3,536	3,650	2,697	2,221	2,764	2,030
Maximum length	4,853	4,348	5,812	4,380	4,401	5,445	7,541	5,979	5,016	5,412	5,049
